# Predisposing Factors and Impact of Child Victimization: A Qualitative Study

**DOI:** 10.3390/ijerph18179373

**Published:** 2021-09-05

**Authors:** Siti Nor Ain Wahid Satar, Mohd Noor Norhayati, Zaharah Sulaiman, Azizah Othman, Lili Husniati Yaacob, Nik Hussain Nik Hazlina

**Affiliations:** 1 Department of Family Medicine, Health Campus, School of Medical Sciences, Universiti Sains Malaysia, Kubang Kerian 16150, Malaysia; sitinorain87@yahoo.com (S.N.A.W.S.); husniati@usm.my (L.H.Y.); 2 Women’s Health Development Unit, Health Campus, School of Medical Sciences, Universiti Sains Malaysia, Kubang Kerian 16150, Malaysia; zaharah@usm.my (Z.S.); hazlinakck@usm.my (N.H.N.H.); 3 Department of Pediatrics, Health Campus, School of Medical Sciences, Universiti Sains Malaysia, Kubang Kerian 16150, Malaysia; azeezah@usm.my

**Keywords:** sex abuse, sex offense, incest, adolescents

## Abstract

Sexual abuse of children is increasing at an alarming rate. This study aims to describe the risk factors and the effects of sexual abuse on children. This unobtrusive qualitative study was conducted on children aged 10 to 18 years old who experienced sexual abuse and followed-up at a psychiatric clinic between the years 2019 and 2021. The information from case records was transcribed. Thematic analysis was performed. Thirty case records were reviewed. The mean age of the victims was 14.6 years; 94% of the victims had experienced vaginal penetration, and 23% of the cases involved incest. The results indicated that socio-psychological predisposing factors involving family structure and dynamic dysfunction, low intrapersonal strength, social influence, and low family socioeconomic status could lead to sexual victimization. This sexual victimization can then lead to emotional turmoil, negative effects on cognitive, academic and social function, negative parental reactions toward the incident, the creation of baby–mother relationships and love–hate relationships, and a lack of goals and hope for the future. Children who experienced sexual abuse may show rape or pregnancy symptoms but may also show entirely non-specific ones. A thorough examination of their history, including biopsychosocial aspects, is necessary to appropriately care for them.

## 1. Introduction

Violence against children is defined as all forms of violence against children below 18 years old, including child maltreatment, physical, emotional or sexual abuse, and neglect by their guardians. Boys and girls are at equal risk of physical and emotional abuse, while girls are at greater risk of sexual abuse [1]. The United Nations International Children’s Emergency Fund (UNICEF) estimated that 15 million adolescents aged 15 to 19 years have experienced sexual abuse in their lifetime [2]. The sexual abuse of a child is defined as any sexual activity conducted with a child who is below the age of legal consent for sexual gratification of an adult or a substantially older child. These activities include oral–genital, genital–genital, genital–rectal, hand–genital, hand–rectal, or hand–breast contact, exposure of sexual anatomy, forced viewing of sexual anatomy or pornography, or using a child in the production of pornography [3]. The World Health Organization classifies child sexual abuse as a silent health emergency [4]. Still, it remains a neglected social issue, especially in cases of incest, even though most reported incest victims are children [5]. Additionally, data from a meta-analysis study showed that victims of intra-familial sexual abuse were younger than the victims of extra-familial abuse [6]. 

There is generally no single factor that results in child sexual abuse. However, research has shown several risk factors to be commonly associated with maltreatment. These include familial dysfunction, divorce, substance abuse by parents, low socioeconomic status, and permissive parenting styles [7]. Research had shown that the cause of child sexual abuse lies with the perpetrator who has psychological problems, which has led them to an inability to control their sex drive and is compounded by a lack of available partners. Hence, it influences the offender’s decision to engage in the behavior [8]. Other factors include early exposure to online chatrooms, which can lead to victims falling prey to perpetrators whom they befriended through the internet [9], and parental mental illness, which approximately doubles a child’s risk of physical or sexual abuse [10].

The short-term and long-term sequelae of child sexual abuse are complex. Major depressive disorder was the most common psychiatric diagnosis, identified in 44.9% of child sexual abuse victims [11]. The longer the first abuse event to the first psychiatric evaluation, the more intense the potential sexual abuse-related psychiatric disorders can be [12]. While the victims may recover, their memories of these experiences may alter their perceptions, thoughts, and emotions, leading to overwhelming feelings of sadness throughout their lives [13].

Cultural taboos prevent Malaysians from discussing sex or sex-related subjects publicly. Individuals who are not sufficiently exposed to accurate sex information are more likely to misunderstand facts and accept myths [14,15], which results in children being unaware or unable to recognize sexual misconduct. Most children suffer in silence because they do not know where to turn for help [5,14], and the abuse lasts for years.

In some cases, the perpetrators force the victims to keep silent, meaning that, even if family members know what is taking place, the victim will not take action due to fear of repercussions [16]. It can have a tremendous psychological impact on children. Accordingly, this study aimed to describe both the risk factors of sexual abuse and its impact on the children who experienced it. 

## 2. Materials and Methods

In this study, we applied the Spaccarelli’s Transaction Model. A qualitative study design was employed to explore the emotions and behavior of children who experienced sexual abuse as recorded by the doctors and social workers who treated them. The unobtrusive qualitative method of data mining did not require the researchers to interact with the respondents of the study; the researchers instead studied the secondary data and extracted relevant information from case records. This was considered appropriate because it allowed researchers to collect data without interfering with the subjects under study, therefore avoiding unnecessary traumatic experiences for these children [17]. 

This qualitative study was conducted in Universiti Sains Malaysia Hospital, Kubang Kerian, Kelantan, located in Peninsular Malaysia. Kelantan state has a population of 1.93 million, and 36.7% of them are children under 18 years old. A retrospective study of children who experienced sexual abuse, whose medical case records were accessible from 2019 to 2021 until current hospital follow-up in Psychiatric Clinic, Universiti Sains Malaysia Hospital (USM), were retrieved. This research obtained approval from the Human Research Ethics Committee of the USM (USM/JEPeM/20110554). 

Children aged 10 to 18 years old who were able to provide information to their doctors were eligible for this study based on inclusion criteria. These criteria were derived based on the children’s age range and their ability to provide information to the treating doctors. Children with vision, hearing, speech impairments, and those with known psychiatric disorders before the abuse, were excluded because these cases were under-reported. This occurred due to the inability of these victims to report their abuse and difficulties in recognizing and understanding the signs on the part of the healthcare services. Purposive sampling was applied, such that hospital case records were deliberately chosen because they could provide the information to the researcher. 

This study was conducted over six months, from January 2021 to June 2021. The case records were skimmed, read, and synthesized until no new information could be obtained [18]. The main source of retrospective data was the case records, which included demographic data, the experience of the abuse, and its emotional, behavioral, education, and social impact on the victims. The list of sexual abuse victims attended to by the Suspected Child Abuse and Neglect (SCAN) team came from the pediatric clinic; this team consists of a pediatrician, psychiatrist, psychologist, social worker, religious officer, and obstetrician and gynecologist. The case records were traced and skimmed to determine their eligibility; relevant information was transcribed using Microsoft Word. The case record extraction guide was used in this study and was described in Table 1, and the focus was on demographic data, abuse history, challenges, and the post-abuse effects on the victims.

The raw data were managed using computer-aided qualitative data analysis software, NVIVO version 12 (QSR International Pty Ltd., Burlington, MA, USA, 2012), and thematic analysis was used to code the information in six phases [19]. All documents were manually read and re-read to get a sense of the victims’ entire story. The researcher coded these transcripts independently and created an initial list of codes, re-evaluated and finalized in consultation with the other researchers. New codes were continuously added throughout this process, and data were constantly compared to the codes to ensure that none of their meanings changed. Similar codes in the victims’ words and the researchers’ interpretations were clustered to develop subthemes; these were then combined into main themes. 

Descriptive analyses were performed using IBM SPSS Statistics version 26.0 (SPSS Inc., Chicago, IL, USA, 2019) for the sociodemographic characteristics of sexual abuse victims. The residential area was determined based on the patients’ addresses as given in the case records; the Kota Bharu district was considered an urban area.

To ensure the rigor of the study, the main themes were agreed upon by all researchers. The saturation of themes occurred when no new themes could be observed in the data. Several validation strategies were also applied as follows. First, any discrepancies in the information were cross-checked with the case records. In the investigator’s triangulation, the co-researchers read selected case reports. Secondly, the thick description specifies the theme by providing many different perspectives to become realistic and richer. Thirdly, negative or discrepant information with contrary information was presented to add credibility to the account. 

## 3. Results

Thirty case records of victims who had follow-up appointments at Psychiatric Clinic Hospital USM between 2019 and 2021 were reviewed. All the victims were females between the ages of 10 and 18, with a mean of 14.6 years old. Table 2 presents the sociodemographic characteristics of the victims, including their family dynamics. In contrast, Table 3 describes the sexual history of the victims, the relationship between the victims and the perpetrators, and the type of sexual abuse they experienced.

### 3.1. Abuse Characteristics 

#### 3.1.1. Perpetrators

All of the child abuse perpetrators in our dataset were men. More than 70% of the victims were sexually abused by a non-relative; of this group, 86% were friends, while foster fathers, employers, and strangers represented 4.3% each; 23% of the cases involved incest; 28.6% of those abusers were brothers; 28.6% were uncles; fathers, stepbrothers, and brothers-in-law represented 14.3% each. Four of the victims were abused by multiple perpetrators, ranging from two to six. The perpetrators’ age was between 15 to 65 years old, and the mean age of the perpetrators was 23 years old. Seven perpetrators were in school, while four were 16 years old or below; all are school dropouts. The other perpetrators either work odd jobs or are self-employed, like fishermen, laborers, and non-government servants. More than half of the victims were sexually abused by their friends or boyfriends as early as three months after knowing the perpetrators. One of the victims, a ten-year-old girl, was raped by a stranger while walking to a nearby shop. One perpetrator had a history of physically abusing his wife and child and then sexually abused his daughter after the wife died. About 50% of the perpetrators had been involved in high-risk behavior, such as illicit substance use, smoking, drinking kratum, addiction to pornography materials, exhibited high libido behavior, and showed a high tendency to molest. 

#### 3.1.2. Types of Abuse

We noted a spectrum of sexual abuse acts ranging from masturbation to vaginal penetration; 6% of the victims were molested, while 94% experienced vaginal penetration. Sexual abuse was associated with physical violence in one case. More than 50% of the victims had consented to sexual activity, and about 40% did not consent to it. However, they were considered statutory rape when the victims were under the age of 16 years. Most of the incidents occurred at night or early morning; the average time of abuse ranged from between 9 p.m. and 3 a.m.

#### 3.1.3. Frequency of Abuse

Repeated episodes of sexual abuse occurred in 90% of cases; the same perpetrators conducted these repeated episodes in 74% of cases. Two of the victims were sexually abused for the first time. Another victim (P16) did not reveal her abuse history, though she presented pregnancy symptoms to the emergency department. 

#### 3.1.4. Circumstances Leading to the Abuse Event

The perpetrator’s visitation or vice-versa became the greatest foreshadower of child sexual abuse, leading to 60% of cases. In contrast, being out on a date led to 17% of cases. Most sexual events occurred after victims went out with the perpetrators or went to the perpetrators’ houses (or vice-versa). Two victims reported that they had quarrelled with their parents beforehand, leading to them sleeping over at their boyfriends’ houses. Some of the victims said that they initially went out with the perpetrators willingly but then were raped. Several victims were raped when their parents went to work, and they were home alone. Child sexual abuse happened in about 20% of cases where the perpetrators and the victims lived together; these cases involved incest. 

#### 3.1.5. Confrontation and Disclosure

In most cases, victims did not disclose the abuse immediately after the event due to fear and threats by the perpetrators, such as, “If you tell anyone, I will kill your brother” (P25). Six perpetrators raped one victim with a learning difficulty (P25) for four consecutive days after being threatened. The victim only confided in her friend, who then informed the victim’s mother. Ten of the victims disclosed the sexual abuse event after experiencing pregnancy symptoms, such as abdominal distension, suprapubic pain, near-fainting, severe nausea, and vomiting; they were then brought to healthcare facilities to confirm the pregnancy. One victim presented with aggressive behavior and a positive drug test; she disclosed the abuse to medical personnel after coming down from the drugs. Some of the victims were forced to reveal their abuse after being caught by family members.

#### 3.1.6. Physical Findings

Physical examination was normal in 96.6% of cases, and signs of physical violence, such as bruises and strangulation marks, were noted in 3.4% of cases; 70% of victims had vulvovaginitis, erythema, a hymenal old tear, and abrasions of the perineum. One-third of the victims were given emergency contraception, either tablet levonorgestrel or combined oral contraceptive pills. Ten percent of victims were prescribed tablet Metronidazole and Doxycycline for two weeks to treat sexually transmitted infections. One victim required examination under anesthesia in the operation theatre because she was too traumatized for standard genital examination. Eight victims came with pregnancy symptoms; pregnancy was later confirmed with urine tests and ultrasonography. 

#### 3.1.7. Behavior during Physical Examination

About 70% of the victims did not show any psychological distress during the interview. They were forthcoming with the interviewers, exhibiting strong eye contact, normal speech patterns, and no psychotic features. About 7% of the victims were not cooperative, and one-third experienced behavioral changes during the assessment. Fear and anxiety were reported in one victim, especially when discussing her family’s reaction to the abuse. The other three victims were sad and worried when discussing the consequences of unprotected sex and sinfulness in terms of religion. From a psychiatric assessment, three victims between 13 to 14 years old exhibited childish behavior, such as holding their breast, yawning, and even smiling during the examination. However, there were signs that some of the questions were only answered by relying on their guardians. In contrast, the other questions were independently responded to by nodding or shaking their heads. 

#### 3.1.8. Post-Disclosure Help-Seeking

After disclosing the abuse, most victims were brought to lodge a police report by family members. The police accompanied about 83% of the victims to the Accident and Emergency Department for a rape kit, while the other 17% came to the facility with labor symptoms. All victims were referred to the SCAN team. Ten victims were found to be pregnant; three of them were moved to a shelter in Kelantan after discovering the pregnancy. The shelter officer brought them for prenatal visits and eventually arranged for the babies’ adoption. After discovering the pregnancy, the other victims stayed with their family members and traveled to health clinics for prenatal visits. They were referred to social services in the district for assessment regarding their home environment and safety.

### 3.2. Themes and Subthemes

Table 4 summarizes the two main themes and subthemes that emerged from the victims’ experiences of sexual abuse and the effects of that abuse as follows: (i) socio-psychological predisposing factors; and (ii) effect of sexual abuse on the victims. 

#### 3.2.1. Socio-Psychological Predisposing Factors

The first theme involves the socio-psychological factors that lead to the victims’ sexual abuse. It is important to understand the psychosocial dynamics present in families in which sexual abuse occurred; this knowledge can lead to prevention and early intervention. 

##### Family Structure and Dynamics

More than 60% of the victims lived with their married parents; two-third of this group lived with both biological parents and one-third with step-parents. Another 36.7% of the victims lived with separated parents, either with their mother or father alone or with a foster family. We found that divorce greatly weakened the relationship between the victims and their parents; one example of a lack of communication and interactions between the victim and her parents can be found in the case of P04.

*Only her mother knew that she was pregnant. Her parents’ relationship was not good. The father did not know that the patient was pregnant despite living in the same house. She had a good relationship with her mother; however, she did not speak to her father for ages. She claimed her father was always angry toward them. *

(P04)

*The parents were divorced in 2008. After that, she lives with her mother and stepfather. Her stepfather raped her at the age of 12, and her stepfather is currently in jail. Since last year, the patient was cared for by her foster family, her father’s best friend. At the age of 15, she had been raped three times by her foster father from June 2019 until February 2020. *

(P08)

After separation, parents remarried then formed complex families with children from previous marriages, breaking the usual practice of a typical family. This complexity within the family leads to one victim getting romantically involved with the perpetrator (a stepbrother), even though they were brought up together. In one case, an unemployed, financially unstable father physically abused his late wife and children and sexually abused the victim after her mother passed away.

*Her parents were divorced when she was a baby; then, her mother married her current father when she was one year old. The stepfather already had twelve children from the previous marriage. They were brought up together since childhood. The patient admitted to having multiple sexual intercourse with her stepbrother from February until April 2020. It was a consensual relationship, and they liked each other. *

(P01)

##### Personal Strength and Interpersonal Relationships

Thirty percent of the victims are introverts and rarely shared their problems with their family members. They kept their secrets among themselves and a small circles of friends. Many mothers described their children as obedient and helpful at home. Two of the victims were over-pampered by their guardians and easily got what they wanted when dealing with financial issues. After they were restricted, they showed rebellious action. More than 50% of the victims had a friendly personality with a big circle of friends. Three of them stated that they were not committed to religious practice in their daily lives. The other two victims said they had been introduced to pornography by the perpetrators before being sexually abused.

*As her grandparents took care of her, the mother described the patient as being over-pampered. She was pampered, rebellious, involved with friends who are rebellious and loitering. She did not pray and was under peer influence. *

(P15)

*According to the sister, the patient was an obedient daughter who occasionally went out and returned home late. She already has a driving and motorcycle license, free to go out on a motorcycle. Premorbid, she was a cheerful daughter but at times hard-headed and pampered by her parents. *

(P28)

##### Social Influences

Our findings noted that the circle of friends was one key factor that affected how the child grew up. For example, two of the victims became involved in illicit drugs after being introduced to them by their friends, and three performed poorly in school after mingling with the wrong company.

*She took Erimin 5 pills a few years ago after being introduced by her cousins but claimed already stopped. The patient had stolen her parent’s money, RM 700, to give to her boyfriend. *

(P05)

*The patient’s mother noted that she was rebellious after mixing with the wrong circle of friends. She always came back late and declined in school performance. She started to rebel when her mother tried to be stricter in finance. *

(P15)

##### Family Socioeconomic Status 

About 70% of the victims’ parents were self-employed, working as rubber tappers, fishermen, laborers, and sellers. Seventeen percent were government servants, and the last 13% of them were unemployed. The families suffered from poor health, including strokes and lack of physical independence; the victims generally experienced low levels of life satisfaction, weak communication, minimal social media-based interactions, and a lack of support from friends and family. A perpetrator financially supported one victim; when the incident happened, her mother ignored it.

*Mother received money from social welfare and payment from a rented house about approximately RM 600 every month. During a shortage of cash, the stepfather only gave a small amount of money. The perpetrator (uncle) usually helped the mother during her financial crisis. After discovering the incident, the mother stopped asking for help from her uncle, but no police report was made to protect her. *

(P30)

#### 3.2.2. Impact of Sexual Victimization Experiences

The second theme involves the effect of sexual abuse on the victims and their parents. The victims may suffer from emotional turmoil, adverse effects on cognitive, academic, and social function, adverse parental reaction toward the incident, the creation of baby–mother and love–hate relationships, and a lack of goals and hope for the future. 

##### Emotional Turmoil and Cognitive Function

Most of the victims showed emotional and behavioral changes due to childhood sexual abuse, usually in anger, embarrassment, sadness, and worry. Most victims felt guilty for disappointing their parents, and some were embarrassed because knowledge of the incident spread throughout their communities; others, however, primarily felt anger toward the perpetrators. Most regretted that they could not prevent the abuse and said they were glad when the perpetrators were caught.

*The patient feels sad now because she embarrassed her mother. She feels guilty to her mother because she did something illegal and not following her religion. *

(P12)

*The patient felt sad and guilty for her actions and was not sure what to do next. She thought of dying but still think about her parents. She did not plan to do anything to harm herself. Feeling sad for making parents sad and ashamed. *

(P18)

*The patient felt remorse for what she did. She knew that sexual intercourse with an unmarried person is a sin. She felt sad about what she did. She claimed to be regretful and shameful after the incident. Plans to be a better person and changed. Unable to contact the accuser afterward. *

(P28)

##### Academic Functioning

The victims’ academic functioning was also affected by a frequent refusal to attend school, a lack of interest in their studies, changing schools, and a reduced attention span. As a result, there is an overall deterioration of academic performance in school. Some of the victims were pregnant and delivered their babies at a young age. After delivery, some babies were adopted, and some of them were taken care of by the victim’s parents. Most of the victims wanted to resume their studies after delivery. A few expressed the desire to change schools due to community knowledge of the abuse. Two of the victims dropped out of school and married their abusers.

*After that incident, she is not schooling and claimed she did not want to marry that man and was not ready to be a mother. She still wants to continue her study after delivery. Father claimed other family members would adopt her baby after delivery. Her family members still accept her and will arrange patient to transfer to another school after delivery. *

(P03)

*She already married in Jun 2020 to the perpetrator. Husband works in a restaurant, and they live with their parents. She is not working and did not go to school after that. *

(P18)

##### Parental Reactions to The Incident

Parental reactions can have significant psychological effects on survivors of child sexual abuse, starting with acknowledging the effects of the incident, such as pregnancy. For parents, incidents of abuse can cause psychological breakdowns and an increase in safety precautions. After the initial shock, most parents became angry, especially after discovering their children’s pregnancies, but eventually accepted it and blamed themselves for not implementing sufficient precautions. One of the parents said that they would be more careful when sending their child to her grandmother’s house, since the perpetrator was her uncle. Another mother said that she had restricted her child’s phone usage in order to keep her from contacting the perpetrator.

*She had a history of sexual intercourse before with her boyfriend last year. Her school teacher informed this after finding pictures on her phone. Since then, the mother has restricted her hand-phone usage. *

(P15)

##### Social Interaction

Sometimes, victims who disclosed their abuse faced bullying and discrimination, whether at home or with peers. This further validates the victim’s internalization of blame and results in the avoidance of social interaction. Additionally, as a cultural norm, speaking about sexual issues is always considered socio-morally bad behavior and is discouraged, especially if the perpetrator supports the family financially. Families also tend to try to keep the knowledge of the sexual abuse from spreading due to a fear of bullying and a loss of power in society.

*She informed her mother after the incident, but no action was taken. She felt angry and sad post the event. The second event occurred two weeks after that. She told the mother, but no action was taken. She did not blame her mother despite no action taken to protect her since her uncle always helps her mother financially. *

(P30)

*Mother wanted to keep this a secret because of fear of backlash and critics from other villagers. She was able to accept what had happened, not blaming or criticizing the patient. She still felt mad at her son. Father is a stroke patient and unable to take any action. *

(P10)

*Since discharge from the ward, the patient is able to cope with the situation, suffer from no nightmare, no recurrent thought about the incident is more focused on online teaching, able to play with siblings. However, the mother is worried that the patient might have trouble coping with the school later on if school starts and the news spread to the whole school. *

(P17)

##### Baby–Mother Relationship

Ten victims became pregnant as a result of the sexual abuse. Of these, three gave their babies up for adoption, three took care of their babies with their families, and two married the perpetrators and cared for the babies themselves. One victim delivered a baby after being raped by her brother; unfortunately, it passed away due to fetal anomalies.

*She delivered a baby but complicated with the imperforated anus, which was done operation for fetal anomalies on day three of life. She wants to continue schooling and look after the baby. She felt love for the baby and expressed her breastmilk in feeding the baby in the intensive care unit. However, the baby passed away on day seven of life. *

(P10)

##### Love–Hate Relationship

The effects of child sexual abuse can affect the victims’ ability to form healthy relationships, damaging their relationships with their parents and families, especially if the perpetrators were a family member.

*Police arrested brother, but his mother bailed him. He was now staying at the same house. He is quiet as usual but spoke to the victim occasionally. She loves his brother but didn’t like what he did. She was able to forgive his brother. *

(P10)

##### Future Hopes and Expectations

Victims discussed their desire to heal, learn coping strategies, move forward, and become better people. In addition, the victims were given sexual education, including information on the definition of sexual intercourse, consequences of unprotected sexual intercourse, such as sexually transmitted infection and unwanted pregnancy, and ways to refuse unsafe or premarital sex. They also discussed their plans for continuing school during their follow-ups. 

## 4. Discussion

The findings highlight some key risk factors for child sexual abuse, including disruption of the family dynamic, low socioeconomic status, and poor peer influences. More than 80% of the victims were of a low socioeconomic class. Most of the victims came from rural areas. Only 15% of their guardians were government servants and had a consistent monthly income. The Fourth National Incidence Study of Child Abuse and Neglect found that low socioeconomic status had a strong relationship with all forms of child abuse, including sexual abuse [20]. One study also reported that most sexual abuse victims came from lower socioeconomic classes [21]. 

One cross-sectional study done in Korea reported that family problems and dysfunctional family dynamics could be associated with child sexual abuse [22]. We found that a pathological and dysfunctional family dynamic contributed to family conflicts, parental violence, less emotional bonding, and the presence of other forms of child abuse like physical abuse and domestic violence. Cases of incest also happened in impaired family dynamics, such as children being adopted by foster families, children who lived alone with their fathers, and children who lived with family members involved in substance abuse. In our study, all of the events of incest were found to be committed by relatives who lived together without consanguinity, primarily fathers, brothers, brothers-in-law, and uncles. More than half of the incest events in our study involved vaginal penetration, while only one case involved the fondling of private parts. 

We found that most victims experienced severe sexual abuse, such as fondling private parts and vaginal penetration, for some time before they disclosed the abuse. This indicated that the victims found it difficult to talk about their abuse due to distress and shame. Sexual abuse is rarely addressed in everyday conversation among family members, even if parents discuss the importance of personal hygiene, developmental issues, or emotional issues with their children. Studies have shown that parents and children rarely talk about sexuality, and when they do, they feel embarrassed [23]. 

One study reported that most of the sexual victimization of adolescents aged between 12 to 21 years within heterosexual romantic relationships occurred within 18 months of the start of the relationship [24]. This finding was similar to our study. We found that the leading causes of child sexual abuse were dating and visitation, where either the perpetrator visited the victims or vice-versa. Smartphones and the Internet acted as the primary communication mediums for the victims and the perpetrators; one study regarding the relationship between internet usage and sexual victimization in college students reported that more than three-quarters of them spent a lot of time with their smartphones and the internet. This is a risk factor for victimization because it can expose potential and can help perpetrators get close to victims [25]. 

Our findings also support a study that has shown that abuse also depends on other factors related to children’s character, family dynamics, community environments, and cultural and social attitudes [26]. The average age of the victims was around 15 years old. Most were in primary and lower secondary school; the younger the child at the time of the abuse, the more complex the disclosure of the event, which can be underdeveloped cognitive, verbal, and recall abilities. We also found that most victims attempted to alert adults to their problems through angry outbursts, serious substance abuse, social withdrawal, school abstinence, or by running away from home. Having a physical disability, especially blindness, deafness, and mental retardation, increased children’s risk of sexual abuse [27].

In our study, 33% of the victims experienced teen pregnancy. Premarital sex and adolescent pregnancy are socially unacceptable in Malaysia and are always kept hidden. In this study, all victims reported premarital sex as rape because the events initially happened against their will. It is considered statutory rape if the victim is less than 16 years old at the time of the event. A review of research on child abuse in Malaysia reported that the mean age of premarital sexual activity was around 15 years old [28]. An increase in premarital sexual activities in younger age groups will significantly increase the risk of teen pregnancies and, later, of abandoned babies [29].

Younger mothers were more likely to have a low socioeconomic status, be single parents, abuse substances, and drop out of school [30]. We noted that most victims confessed that they had no idea what to do; children who experience sexual abuse tend to hide from telling anyone about it because they feel guilty. They are also afraid of the perpetrator’s anger and are sometimes even threatened by the perpetrator. A review of 45 studies on the impact of sexual victimization among children reported that sexually abused children had more symptoms of fear, post-traumatic stress disorder, sexualized behaviors, and poor self-esteem than non-abused children [31]. Another study reported that children are more traumatized when the perpetrators are their parents [32]. 

We also found that child sexual abuse can significantly affect the family environment due to societal and cultural embarrassment. Families feared being labeled “bad” because they failed to protect their children. In addition, many Asian communities are embarrassed to discuss sexual abuse because of a fear of public exposure and a fear of culturally insensitive responses from professionals [33]. Culture, therefore, plays a significant role in evaluating the victim’s symptoms and potential outcomes. When a case of child sexual abuse is reported, a psychiatrist must explore the child’s family dynamics, particularly their religious understandings and belief systems, in addition to clinical assessments. This necessary information includes the child’s knowledge of the sexual activity, the reasons for the abuse, the victim’s relationship with the perpetrator, the process of disclosure, the support system, and coping strategies. 

When summing up all the risk factors from our findings and their effects on child sexual abuse, we applied Spaccarelli’s Transactional Model [34] to understand why children react differently to different stressors. In this model, sexual abuse is the stressor that creates a risk of maladaptive adjustment; the level of risk depends on the level of abuse-related stress. It can be mediated by other factors, such as support systems, coping mechanisms, and a child’s cognitive appraisal. This model also offers a comprehensive approach for developing intervention methods for the victims experiencing post-traumatic stress disorder.

### Limitations

This qualitative study explored data from case records, limiting the exploration of the victims’ emotions, and hence, there was the possibility of recall bias. In-depth interviews with victims could lead to important information for the clinicians, and the clinicians could explore this with more specifications. This study also excluded children with disabilities, who will have more intrusive abuse experiences. We cannot explore their experience of sexual abuse because children with disabilities may have limitations in speech and language and require specific tools such as photos or drawings. Children with disabilities may only answer yes or no when answering the question during the consultation and may limit the researchers in an exploration of their history.

## 5. Conclusions

All victims in this study complained of sexual abuse, but it can present non-specific behavioral and physical symptoms, including school refusal, sleep disturbances, or chronic symptoms like headache or stomach ache. It is necessary to take a history from the children to diagnose and determine the appropriate tests, treatments, and potential need to report suspected child sexual abuse to child protection and law enforcement services. If they have not disclosed the abuse, these children should be questioned carefully and in a non-leading manner about the possibility of sexual abuse. Child sexual abuse dramatically affects the victim and society; it cannot be ignored, and prevention and intervention programs must be implemented. School-based awareness and educational programs should be promoted in Malaysia to keep children informed of the potential risks.

Studies on the long-term effects of sexual abuse on survivors are required. They need to consider the implications of child sexual abuse on their eventual parenting styles; many survivors may not realize that some traumas may interfere with their parenting practices. There is a need to evaluate further the effects of child sexual abuse on survivors’ conversations with their children about sex to determine whether these mothers could benefit from a parenting intervention to help them engage in healthy discussions with their children.

## Figures and Tables

**Table 1:** Case record data extraction guide.

Domains	Information of Interest
Demographic Information	How old is the victim? Where did the victim live? With whom the victim lived at home? How about the victim’s education level?
The Abuse History	What has happened to the victim that led him/her to be in Social and Welfare Department custody? How old was the victim when it happened the first time? Who is the perpetrator? A family member or an outsider? What is the sex of the perpetrator? How did the victim know the perpetrator? Online/smartphone? What is the nature of the assault (anal, vaginal and/or oral penetration)? How long has the abuse lasted? Are there any forces involved? Did it happen one time or more than one time? Did the abuse happen during the day, at night, or both?
The Challenges	How difficult is it for the victim to go through it? How did the victim disclose the event? Is it purposeful or accidental (any physical changes or pain)? Whom does the victim talk to? What did the victim do to forget about it? Did the victim feel threatened or in danger or embarrassed/shameful/fearful? Is it associated with emotional distress (eating or sleeping disturbances, aggressive or withdrawn behaviors)? What are the blood results for infective screening? Who brought the victim for a follow-up? How about the support system?
The Impact	What did the victim expect for his/her future? What was the victim’s wish? Get married or continue studying? Did the victim need long-term psychiatric follow-up?

**Table 2:** Sociodemographic characteristics of sexual abuse victims (*n* = 30).

Variable	Mean (SD)	n (%)
Age (years)	14.6 (1.77)	
Race		
Malay		29 (96.7)
Non-Malay		1 (3.3)
Highest education level		
Primary school		3 (10.0)
Lower secondary school		18 (60.0)
Upper secondary school		9 (30.0)
Residential area		
Rural		28 (93.3)
Urban		2 (6.7)
Parental status		
Married family		19 (63.3)
Separated parents family		11 (36.7)
Living arrangement		
Living with both parents (biological or step)		19 (63.3)
Lived with mother alone		9 (30.0)
Living with father alone		1 (3.3)
Living with foster family		1 (3.3)
Main guardian occupation		
Unemployed		5 (16.7)
Self-employed		21 (70.0)
Government servant		4 (13.3)

**Table 3:** Sexual history of the victims.

ID	Age	Type of Sexual Act	Suspected Perpetrator (Age)	Circumstances of Sexual Abuse Happened	Abuse (Most Serious)
P01	14	Consensual rape	Step brother (17)	Live together	Vaginal intercourse
P02	16	Consensual rape	Boyfriend (23)	Dating	Vaginal intercourse
P03	16	Consensual rape	Boyfriend (20)	Visitation	Vaginal intercourse
P04	15	Consensual rape	Boyfriend (19)	Visitation	Vaginal intercourse
P05	14	Non-consensual rape-2 perpetrators	Friends (22) and (18)	Dating	Vaginal intercourse
P06	13	Non-consensual rape-3 perpetrators	Boyfriend and his friends (17–20)	Dating	Vaginal intercourse
P07	15	Consensual rape	Boyfriend (16)	Visitation	Vaginal intercourse
P08	17	Non-consensual rape	Foster father (40)	Living together	Vaginal intercourse
P09	15	Non-consensual rape	Brother (24)	Living together	Vaginal intercourse
P10	14	Non-consensual rape	Brother (15)	Living together	Vaginal intercourse
P11	16	Consensual rape	Brother in law (26)	Living together	Vaginal intercourse
P12	15	Consensual rape	Boyfriend (15)	Visitation	Vaginal intercourse
P13	15	Consensual rape	Boyfriend (16)	Visitation	Vaginal intercourse
P14	16	Molestation	Employer (28)	Dating	Masturbation/ejaculation
P15	16	Consensual rape	Boyfriend (20)	Visitation	Vaginal intercourse
P16	16	Rape	Not revealed	Not revealed	Not revealed
P17	10	Non-consensual rape	Stranger (20)	Dating	Vaginal intercourse
P18	15	Consensual rape	Boyfriend (19)	Visitation	Vaginal intercourse
P19	10	Molestation	Uncle (42)	Visitation	Fondling private parts
P20	13	Consensual rape	Boyfriend (17)	Visitation	Vaginal intercourse
P21	12	Non-consensual rape	Brother’s friend(17–18)	Visitation	Vaginal intercourse
P22	14	Consensual rape	Boyfriend (16)	Visitation	Vaginal intercourse
P23	16	Non-consensual rape	Friend (27)	Visitation	Vaginal intercourse
P24	17	Consensual rape	Boyfriend (19)	Visitation	Vaginal intercourse
P25	13	Non-consensual rape—6 perpetrators	Friend and strangers (18–22)	Visitation	Vaginal intercourse
P26	14	Non-consensual rape	Father (40)	Living together	Vaginal intercourse
P27	15	Non-consensual rape	Brother’s friend (18)	Visitation	Vaginal intercourse
P28	17	Consensual rape	Boyfriend (23)	Visitation	Vaginal intercourse
P29	14	Non-consensual rape—4 perpetrators	Boyfriend and strangers	Visitation	Vaginal intercourse
P30	15	Non-consensual rape	Uncle (65)	Visitation	Vaginal intercourse

Note: Consensual rape: victim agreed and gave consent before sexual activity; Non-consensual rape: victim was forced into sexual activity; Dating: abusive, controlling, and aggressive behaviors occurring in a current dating relationship can take the form of sexual abuse; Visitation: the sexual abuse happened when the perpetrator came to the victim’s house or vice-versa; Living together: the sexual act occurred between people living in the same house.

**Table 4:** Themes and subthemes.

Themes	Subthemes
Socio-psychological predisposing factors	i. Family structure and dynamic ii. Intrapersonal strength iii. Social influence iv. Family socioeconomic status
Impact of sexual victimization	i. Emotional turmoil and cognitive function ii. Academic functioning iii. Parental reactions toward the incident iv. Social interaction v. Baby–mother relationship vi. Love–hate relationship vii. Future hope and expectations
